# The role of dietitians and physiotherapists in the prevention of non-communicable diseases in Italian communities: lessons from orthopaedic care to strengthen community initiatives

**DOI:** 10.3389/frhs.2025.1634923

**Published:** 2025-07-18

**Authors:** Matteo Briguglio, Thomas W. Wainwright, Claudio Cordani

**Affiliations:** ^1^ Laboratory of Nutritional Sciences, IRCCS Ospedale Galeazzi—Sant’Ambrogio, Milan, Italy; ^2^ Orthopaedic Research Institute, Bournemouth University, Bournemouth, United Kingdom; ^3^ NHS Foundation Trust, University Hospitals Dorset, Bournemouth, United Kingdom; ^4^ Lanzhou University, Lanzhou, Gansu, China; ^5^ Department of Biomedical, Surgical, and Dental Sciences, Università degli Studi di Milano, Milan, Italy; ^6^ Laboratory of Evidence-Based Rehabilitation, IRCCS Ospedale Galeazzi – Sant'Ambrogio, Milan, Italy

**Keywords:** health care facilities workforce and services, osteoarthritis, orthopedics, healthy eating, physical activity, population program specialist, combined modality therapy, quality of health care

## 1 From reactive to preventive care model

Non-communicable diseases (NCDs), often referred to as chronic diseases due to their tendence of being of long duration, arise from a multifactorial interplay of genetic, physiological, environmental and behavioural determinants. Globally, NCDs continue to increase in both prevalence and impact, accounting for nearly 80% of all years lived with disability before the age of 70, and contributing to the premature death of approximately 50,000 individuals per day in 2021 (1). In addressing the burden and chronicity of NCDs, prevention strategies ought to constitute a central pillar of clinical and public health practice. Among these, the promotion of healthy lifestyles may play a pivotal role, as such behaviours directly influence modifiable determinants of health at both individual and population levels. Health behaviours that should be promoted and that are widely recognised for their benefits include maintaining a balanced diet, engaging in regular physical activity, ensuring sufficient sleep, and abstaining from smoking and alcohol consumption (2). The failure to adopt these lifestyle principles can substantially increase the risk of developing and aggravating of NCDs, as in other pathologies. This is because inadequate nutrition, sedentary behaviours, sleep deprivation, tobacco use, and alcohol intake impair immune function, diminish body's reserves, reduce intrinsic capacity, compromise functional ability, and erode resilience (3, 4). Consequently, nutritional disorders such as undernutrition and obesity, cardiovascular diseases such as hypertension, respiratory diseases such as chronic obstructive pulmonary disease, metabolic disorders like diabetes, and musculoskeletal conditions such as osteoarthritis and sarcopenia are likely to develop and progress. The clinical management of these conditions typically requires considerable healthcare resources and may impose a significant physical and mental burden on affected patients (5).

A shift in health policy towards community-based health promotion ought to be prioritised, as such a transition would allow to address the problem at its source. This is now steered globally, as exemplified by the project “Joint Action Prevent Non-Communicable Diseases” (https://preventncd.eu). To design effective public health policies, a transition from hospital-centred to community-based models of care must occur. In Italy, the urgency of this shift is underscored by the high prevalence of chronic diseases, which affects 45% of subjects across all age groups and up to 60.8% of individuals aged over 65 (6). In response, Italian Ministerial Decree number 77 of 2022 (7) initiated a policy reform aimed at establishing new models and standards for the development of multidisciplinary territorial assistance throughout the country.

Despite the progress made in hospital-based multimodal care, the expansion of allied health professions' involvement in community prevention and health promotion remains underdeveloped. This opinion paper seeks to articulate the emerging roles of two key health professionals, i.e., dietitians and physiotherapists, who are particularly well-placed to address modifiable risk factors such as poor dietary habits and physical inactivity. Furthermore, we propose the integrated orthopaedic care pathway as an illustrative model, highlighting how interprofessional collaboration may foster holistic, preventive approaches consistent with the evolving framework of the Italian National Health System (NHS).

## 2 Dietitian as champions of community nutrition

In the Italian healthcare context, the registered dietitian nutritionist is formally recognised as an allied health professional with specialised expertise in all activities aimed at the correct application of food and nutrition, as defined by Ministerial Decree number 744 of 1994 (8). In the context of community-based care, dietitians play a fundamental role in addressing suboptimal dietary behaviours, which are widely acknowledged as leading modifiable risk factors for chronic disease, and in promoting population health more broadly. Their public health competencies may be categorised into two key domains: (1) the provision of dietary and nutritional assistance, encompassing both clinical and educational dimensions, and (2) collaboration in the development and implementation of inclusive food policies responsive to the health needs of communities. In this regard, community dietitians ought to serve as “champions” of nutritional health, leading initiatives in education, counselling, and public health advocacy.

Nutrition education, often conceptualised as nutritional literacy, incorporates a range of context-specific strategies, including culturally adapted school meal plans, cooking demonstrations, nutrition awareness activities in workplaces, weight maintenance support in public counselling services, and education regarding food safety, ethics, and sustainability. These practices may be directed at the general public or targeted subpopulations and are critical in promoting health-conscious decision-making. Dietitians also undertake individualised nutrition counselling, which involves targeted, evidence-informed dialogue with individuals requiring a personalised approach, such as those with obesity, hypertension, or type 2 diabetes. Beyond education and individual counseling, dietitians may significantly influence public policy through nutrition advocacy. These activities are typically informed by practical experience, nutrition surveillance, and epidemiological data concerning dietary patterns and consumption habits at the community level. For instance, dietitians might establish partnerships with media and governmental or private institutions to co-develop communication campaigns. Such initiatives should aim to maximise outreach while continuously evaluating and refining messaging based on feedback and impact assessment (9).

Collectively, these competencies position dietitians as valuable assets in community health systems, particularly in settings where population with varying levels of health coexist. In such contexts, the capacity to design and deliver interventions across the spectrum of prevention (primary, secondary, and tertiary) must be regarded as essential.

## 3 Physiotherapists as champions of community rehabilitation

In Italy, the registered physiotherapist is the allied healthcare professional with expertise in the prevention, treatment, and rehabilitation of motor skills, higher cortical functions, and visceral functions, across a broad spectrum of pathologies. This definition and professional scope are governed by Ministerial Decree number 741 of 1994 (10). From a public health perspective, physiotherapists are entrusted with two principal responsibilities: (1) the promotion of health in its biopsychosocial dimensions to protect and support vulnerable populations, and (2) collaboration in the development and implementation of care and rehabilitation pathways that ensure equitable access to safe and effective services for all citizens.

In community settings, physiotherapists ought to be regarded not only as rehabilitation specialists but also as key contributors to proactive, preventive health strategies. Their work may include patient education aimed at conveying understanding of the cardiorespiratory and neuromuscular benefits of regular physical activity and supporting the adoption of exercise routines beneficial to maintaining health. These interventions should be personalised, for instance, according to the type of musculoskeletal disorder, the degree of pain experienced, or the goal of improving movement efficiency. Such personalisation is particularly pertinent when addressing sedentary behaviours, which are key contributors to the development and progression of NCDs. In these cases, physiotherapists can play a preventive role by designing interventions that may help maintain functional autonomy and delay the onset of disability considering important determinants of health such as home autonomy and the presence of family support. Beyond direct patient care, physiotherapists may also engage in health policy advocacy aimed at strengthening rehabilitation services and promoting their integration into broader healthcare systems (11). Examples of such contributions include efforts to streamline service delivery in order to optimise the use of available resources, minimise wasteful activities, and prioritise rehabilitation pathways and means based on prevailing needs of the community and to reduce environmental impact (12).

These competencies make physiotherapists vital assets within community-based health systems, particularly in their ability to encourage environments that promote independence and mitigate the risk of disability-related social exclusion or marginalisation. In these settings, physiotherapists should be integrated into multidisciplinary teams working collaboratively to ensure continuity of care and equitable access to preventive and rehabilitative services.

## 4 Alignment with Italian healthcare reform

The Italian healthcare system is currently undergoing a structural reorganisation, shifting from a hospital-centred model towards a community-based network of services. This transformation is strategically supported by the National Recovery and Resilience Plan (Piano Nazionale di Ripresa e Resilienza, PNRR). The reform introduces new healthcare delivery frameworks, such as Case della Comunità (Community Houses), intended to provide proximity care through integrated services. These reforms also picture expanded scopes of practice for allied health professionals, including the family and community nurse, who may deliver nursing care across various levels of clinical complexity. This reconfigured territorial care model, which includes the establishment of other health services (7), is designed to ensure continuity across prevention, chronic disease management, and interprofessional collaboration. In this evolving landscape, dietitians and physiotherapists, by virtue of their academic preparation, clinical training, and regulatory standing, should be regarded as key actors in strengthening community-based prevention efforts. Their established expertise in medical nutrition therapy and exercise therapy across the lifespan, respectively, position them ideally to contribute to public health education, lifestyle counselling, and advocacy.

Historically, the hospital-centred approach has shaped the practice of these professions, focusing on service efficiency, accessibility, quality, and sustainability. However, while the legal definitions of these roles, as outlined in the 1994 Ministerial Decrees, remain in place, the shift in organisational priorities now presents an opportunity to extend their scope. These professionals ought to be more fully integrated into multidisciplinary community teams where they may address modifiable risk factors such as poor diet and physical inactivity through person-centred, evidence-informed strategies. Community Houses could serve as centres where dietitians deliver group-based nutritional literacy sessions and counselling interventions, while physiotherapists facilitate the adoption of disease-specific exercise routines and functional mobility programmes. Looking ahead, an effective model of primary care might include the formal presence of a “family dietitian” and a “family physiotherapist” associated with each general practitioner. Considering that a general practitioner in Italy may serve up to 1,500 patients, task delegation related to preventive care could substantially alleviate their clinical burden. Similarly, paediatricians, who manage large and diverse caseloads, could benefit from the support of paediatric dietitians and physiotherapists. These professionals may also assist families in understanding how to sustain optimal nutrition and physical activity practices throughout childhood and adolescence, thereby enhancing preventive care and developmental outcomes.

Internationally, primary care models vary in their degree of integration and maturity. As highlighted in a recent systematic review evaluating multidisciplinary management of chronic conditions in non-hospital settings (13), team-based approaches may improve patient-reported outcomes, although further evidence is needed to confirm clinical effectiveness. In Italy, the incorporation of physiotherapists into primary care teams has only recently begun in certain regions, where they are involved in early physical activity interventions and patient education. In contrast, the United Kingdom has already institutionalised this model through the “first contact practitioner” role, which facilitates timely access to musculoskeletal care. The expansion of allied health professionals' scope of practice may enhance patient empowerment and support preventive efforts across the primary, secondary, and tertiary continuum (14), complementing the roles of general practitioners and community nurses. Countries such as Canada (Family Health Teams) (15), Australia (Healthy Together Victoria) (16), and Denmark (Health Promotion Packages) (17) provide additional examples of interprofessional collaboration in primary care.

In the context of PNRR implementation, the need to recognise the roles of all health professions, including dietitians and physiotherapists, in the delivery of care, prevention, education, and innovation has been underscored by the National Federation of Orders (FNO) of Medical Radiology Technicians (TSRM) and of the Technical Health Professions for Rehabilitation and Prevention (PSTRP) (18). The Federation's position document calls for inclusive decision-making processes and the dismantling of siloed service models. It advocates for multidisciplinary teams as the operational core of Community Houses and home-based care and emphasises the expansion of screening and prevention services across the life course, the integration of telemedicine, the enhancement of data interoperability, the broadening of essential levels of care and social support, the modernisation of health education programmes, and the prioritisation of evidence-based innovation.

## 5 An example from orthopaedic patient care

Although orthopaedic care is predominantly delivered within hospital settings and is not traditionally associated with primary prevention, it may nonetheless serve as an illustrative model of how multidisciplinary, low-intensity strategies, particularly those implemented during the prehabilitation and post-rehabilitation phases, can inform scalable, upstream preventive approaches. This model suggests that insights derived from orthopaedic care might be extrapolated to broader chronic disease prevention and health promotion frameworks within community contexts.

The orthopaedic pathway may be conceptualised in four distinct yet interconnected phases: prehabilitation, perioperative care, rehabilitation, and post-rehabilitation. Each stage involves the coordinated contribution of a multidisciplinary team, including the ERAS (Enhanced Recovery After Surgery) nurse, physiotherapist, dietitian, speech pathologist, and dental hygienist, all of whom operate across the continuum of care (19). Prehabilitation, in particular, exemplifies a proactive, prevention-oriented strategy. It seeks to mitigate preoperative risks and enhance postoperative recovery through a variety of interventions, including the promotion of a healthy and balanced diet, cessation of smoking and alcohol consumption, exercise therapy calibrated to the patient's preserved intrinsic capacity, and oral hygiene screening aimed at preventing oral disease-associated periprosthetic joint infections. These initiatives could be directly transferable to models of prevention for NCDs such as obesity and diabetes, where early lifestyle modification is essential. Rehabilitation commences following discharge from the surgical department and may be undertaken either in specialised facilities or within the patient's home environment. The objective of this phase is to restore functional capacity by means of a disease-specific, personalised exercise regimen, dietary support, and, in cases of maxillofacial surgery, targeted interventions for chewing and swallowing rehabilitation. The post-rehabilitation phase, which has historically received limited attention, has more recently come to be recognised as a critical period for reinforcing health education, supporting patient self-management, preventing relapses and falls, and sustaining long-term adherence to healthy behaviours (20). As such, it represents a valuable opportunity for ongoing engagement with patients to consolidate gains made during earlier stages of care.

Beyond its relevance to the orthopaedic context, the structured integration of low-intensity interventions within territorial and home-based settings, particularly in the prehabilitation and post-rehabilitation phases, aligns closely with the preventive and essential care services envisioned under the evolving Italian NHS. The early involvement of patients in care planning, multi-level collaboration among professionals, and interprofessional synergies were recently appraised for their potential public health benefits. These include the prevention of conditions leading to surgery, the mitigation of disability, the enhancement of physical and nutritional resilience, the promotion of independence and self-management in activities of daily living, and the improvement of long-term quality of life (20, 21). Digital tools such as tele-nutrition and tele-rehabilitation platforms, initially proposed and more recently adopted for remote patient monitoring in specialist orthopaedic settings (22, 23), may also be adapted to broader preventive care models. These technologies could be seamlessly integrated into territorial and home telemedicine services, thereby enhancing care delivery, promoting early intervention, and contributing to the development of more comprehensive and interoperable patient data systems.

## 6 Conclusion

The increasing demand for complex care, combined with rising expectations regarding quality of life, will likely constitute key challenges for future global health systems. Substantial benefits could be realised by prioritising prevention over treatment. It is now well established that NCDs are largely influenced by behavioural factors. A coordinated strategy, centred on healthy eating and physical activity and scaled across multiple levels of care, may significantly impact both individual and population health outcomes (5, 24). Within this framework, community dietitians and physiotherapists should be recognised as pivotal advocates for advancing a prevention-oriented healthcare paradigm in Italy. These professionals ought to embrace a dual mandate: not only to provide therapeutic management of acute conditions for which they are consulted (e.g., undernutrition, reduced physical function, or joint pain), but also, where appropriate, to promote sustainable and health-enhancing behaviours. The community care pathway could draw inspiration from the orthopaedic care model, wherein a multidisciplinary team delivers holistic patient management, complemented by remote care services. This model may serve as a compelling example of integrated, non-siloed care. By applying similar strategies within community settings, particularly for individuals at risk due to modifiable lifestyle-related factors, the onset or escalation of disease, and in some cases, the need for surgical intervention, might be delayed or entirely prevented. Embedding dietitians and physiotherapists into territorial care infrastructures would represent a meaningful step towards realising a healthcare system that is not only curative, but increasingly preventive, equitable, and resilient.

### 6.1 Future directions

In the near future, the development of a reoriented Italian NHS should be guided by several strategic priorities. These include: (1) the harmonisation of dietitians' and physiotherapists' roles across all levels of territorial assistance; (2) the formal establishment of essential levels of nutrition and rehabilitation services; (3) the integration of care models for orthopaedic patients requiring home-based support both pre- and post-operatively; and (4) the consolidation of tele-nutrition and tele-rehabilitation services to ensure advanced, continuous care during the hospital-to-community transition.

Furthermore, it is imperative to anticipate and address the challenges that may accompany the shift from reactive to preventive care. Barriers such as increased workload, time constraints within clinical appointments, insufficient reimbursement for preventive services, and the absence of clearly defined care pathways must be recognised and mitigated through policy, education, and system-level reform (25). A forward-looking health system must therefore be proactive in supporting professionals through improved structures, clearer guidance, and sustained investment in preventive health infrastructure. In doing so, Italy may foster a more sustainable and person-centred healthcare model, that is, one capable of addressing the multifaceted demands of chronic disease prevention and health promotion across the life course.
